# Annotations

**Published:** 1891-03-14

**Authors:** 


### Annotations.

During the last ten years the North of London has been making strenuous efforts to supply itself with adequate hospital accommodation. In the Holloway Road there is a new building, which though occupied and treating last year about a thousand in-patients and more than fifteen thousand out-patients, cannot be completed for want of money. The building contains sixty beds, and not only are those sixty practically always occupied, but "shake-downs" and other temporary arrangements for increasing the accommodation are constantly in use. At the recent annual meeting of the governors it was stated that owing to the want of adequate accommodation, a great many serious cases had either been sent to other hospitals or had been obliged to wait a long time before they could be received. All this we know of our own knowledge to be true, and, in fact, very much within the truth. The hospital in the Holloway Road, the Great Northern Central, contains sixty beds. If it contained six hundred it would not be too large for the vast district which it supplies, and in which it is practically the only available medical charity. It is a well-managed hospital, and has a thoroughly trustworthy and attentive committee. If there be a fault to be found it is, perhaps, this, that the committee, as a body, are somewhat too timid, and the members of the medical staff a little wanting in appreciation of the general practitioners of the neighbourhood. These, however, are not faults which impair the usefulness of the hospital; though if they were removed, there is no doubt that the institution would be much more popular in the neighbourhood. It is eminently desirable, that the interests of the sick poor should be considered before all things in this connection; and those interests demand the immediate completion of the hospital. When it is completed it will give accommodation to twice the number of patients it now serves, and it can be completed for £20,000. The committee have in hand about £1,000 towards this considerable sum: they want £19,000 more. If we knew exactly what words to say to persuade the public to give the committee that sum, we would say them here and now. As it is, we can only assure our readers that the needs of the district are at least ten times greater than its hospital resources; that the Great Northern Central is doing its best to overtake those needs; that it is a well-managed institution; and that it well deserves all the help that can be given to it.

**More Hospital Accommodation for North London.**

The Medical Officer for the Borough of Huddersfield (Dr. James R. Kaye, M.B., D.P.H.) has forwarded to us a circular letter which it is intended to issue to all those families who prefer to treat cases of infectious disease at home rather than to send them to hospitals. The subject is of great importance. The majority of well-to-do people shrink from sending their sick children or other members of the family to hospitals when ill with fevers. We all feel as if it were a heartless kind of thing to do. As a matter of fact it is not: on the contrary, it is often the very kindest step that can be taken. But whatever abstract reason may say, feeling comes in and insists upon being heard: and feeling prevails upon us to keep our sick ones at home if we can by any means afford to do so. Under these circumstances, practical instructions given by recognised authorities are of great value; and the prosperous classes of Huddersfield ought to be very grateful to Dr. Kaye for the simple and judicious instructions he has prepared for their use. The instructions are set forth under the heads of "Preparation of Room," "Management of Sick Room," "Nursing," "Medical Treatment," and "Final Disinfection." With regard to the preparation of the sick room, Dr. Kaye says that, other things being equal, the uppermost storey of the house is to be preferred, because a room or rooms on that storey can be most easily and completely isolated. The room should possess the four indispensable requisites of being large, light, airy, and cheerful. All unnecessary furniture of every kind, more particularly carpets and curtains, should be taken away; and every drawer of the apartment should be emptied and its contents removed from the room. Generally speaking, the barest apartment is the safest apartment for a case of fever. Under the head of "Management," Dr. Kaye insists that a sheet well moistened with disinfectant fluid should be hung over the doorway. Here it is important to note two things: first, that the sheet must be large enough not only to cover the whole of the doorway, but to overlap it at least three or four inches on every side; and, secondly, a corner of the sheet should be constantly immersed in a vessel containing some of the disinfectant. "Keep a moderate fire always burning" in a sick room, says Dr. Kaye. And why? Because the best of all ventilators in a room is an open chimney and a fire in the grate. "Open the window," insists the doctor. But here again the window is to be opened with intelligence, and not with stupidity. Open it at the bottom. But take care to have a piece of wood so accurately made as to fit exactly into the opening thus made at the bottom of the window. It is not intended that air shall enter there and kill the patient and the nurse with draughts. When the lower sash is raised three or four inches, an opening is made between the two sashes at the middle of the window, and there the air is to enter. But why is the air to enter at the middle? Because when the lower sash is raised—but the opening closed—the air can only penetrate the room in an upward direction; the current of cold air thus passing straight up towards the ceiling is gradually diffused among the hot air at the top of the room, and draughts are effectually prevented. With a window thus opened, and a moderate fire in the grate, ventilation is constant and complete. Under the head of "Nursing," Dr. Kaye's direction may be summed up in a single sentence: "Nurses! Carry out the doctor's orders faithfully." "The treatment," too, must, of course, be left to the doctor. Under the head of "Final Disinfection," brief, but clear and sufficient instructions are given. The particular disinfectants preferred by Dr. Kaye are Condy's fluid, carbolic acid, St. Bede's disinfectant, chloride of lime, and others of the same class.

**Sanitation at Huddersfield.**

The *Nightingale* does us the honour to reproduce our article of February 7th on "Medical Women at the Johns Hopkins University," and to offer some comments thereupon. The writer in the *Nightingale* partly avoids the main question at issue, and yet makes an effort to defend the position whilst shrinking from a frank avowal of it. The point at issue is not that American women have asked for admittance at the Johns Hopkins University. If it had been this and nothing more we should have said, admit them by all means, and provide for them instruction in every respect as full, complete, and thorough as you provide for men. But, if we are not misinformed, what the medical women have asked for is that they shall be admitted to the Johns Hopkins University "on the same terms as men." If we understand this aright it means that women are to study along with men throughout the whole medical curriculum, and that they are to be regarded by their teachers, fellow-students, themselves, and all concerned as if they were of the same sex as men. If this be so, and if it be a result brought about by the sole action of American women, we are deeply and sincerely sorry for those women. The writer in the *Nightingale*, by way of defence, gives the instance of a nurse of twenty-two who witnessed her first obstetrical case on night service in the company of a young Interne of the same age. The same nurse at twenty-four years of age had a private case alone, except that a young man was in attendance, and in a suite of rooms in a large American hotel. That this young woman was a pure-minded girl, following a lofty ideal of duty, and unmoved by accidental circumstances, we are the last to wish to deny. We should hold ourselves debased and degraded by suggesting any insinuation to the contrary. But these are among the very experiences which girls of such immature years should not be called upon to meet. They ought not to be possible. Man is man, and woman is woman, and nature has put a veil between them; a veil which should never be torn through. We cannot, perhaps, explain in hard, mechanical terms exactly what we mean. But there is feeling in every chivalrous man's heart that woman, especially if she be young, should be hedged round with a kind of divine and protecting mystery. We do not care to argue the point at length; we enter our protest, and we feel assured that if all the cultur ed men of Great Britain, and the uncultured men too, could be polled, nineteen in every twenty of them would stand with us, and say that to train girls with young men in such studies as anatomy, physiology, and medicine is to rob them of that rare delicacy which is one of the crowning endowments of the sex, and to inflict upon them an injury which will be lifelong and irreparable.

**A Question of Propriety.**

